# Prevalence and correlates of sexual harassment in professional service firms

**DOI:** 10.3389/fpubh.2022.1082088

**Published:** 2023-01-20

**Authors:** Kris Hardies

**Affiliations:** Department of Accounting and Finance, University of Antwerp, Antwerp, Belgium

**Keywords:** sexual harassment, gender harassment, workplace harassment, workplace sexual violence, professional service firm, professional service firms, accounting firms, law firms

## Abstract

**Background:**

Despite their significance, limited research has been conducted on sexual harassment in professional service firms (PSFs).

**Methods:**

Survey data were gathered from 321 Belgian employees (155 women, 166 men) of global accounting and law firms. The prevalence of sexual harassment in PSFs based on traditional sexual harassment items and not-man-enough harassment items was reported. Correlations of respondents' experiences with workplace sexual harassment with perceived acceptance of sexual harassment by one's peers (social norms), personality traits, and demographic and job-related factors were examined.

**Results:**

Experiences of workplace sexual harassment were widespread in the current sample: 88.5% of women and 83.3% of men experienced at least once or twice some form of sexual harassment at work during the past 24 months. The most frequent types of sexual harassment reported are examples of (verbal) forms of gender harassment. Instances of traditional harassment were experienced equally often by men and women, while not-man-enough harassment was much more frequently experienced by women. Severe physical sexual harassment was less frequent. Workplace sexual harassment is positively correlated with perceived acceptance of sexual harassment by one's peers and negatively with job level.

**Conclusions:**

Results of the current study align with research that links workplace sexual harassment with workplace culture and suggest that both men and women in PSFs experience enforcement of gender roles. It seems quintessential for firms to create working environments and cultures in which sexual harassment is clearly and unambiguously condemned and sanctioned.

## 1. Introduction

Sexual harassment is a tremendously important topic because of its prevalence and impact. Sexual harassment refers to a wide range of unwelcomed sex and gender-related behaviors, ranging from unwanted sexual actions and assaults to sexual propositions and requests, sexual comments, and non-verbal sexual gestures. Existing reviews indicate that 40%−75% of women and 15%−30% of men experience potentially harassing behaviors at work (examples include being called insulting names, getting inappropriate remarks about looks, being a victim of stereotyping and prejudice, being stared at, being touched unnecessary and unwanted) (1–3). Such experiences have various adverse effects on people's lives, including job satisfaction, job productivity, and psychological and physical wellbeing (4–6). Moreover, sexual harassment affects not just the involved victims; it also negatively affects people witnessing such harassment (7, 8) and organizations at large (6, 8).

The current study reports on the prevalence and correlates of sexual harassment in professional service firms (PSFs). Despite their significance, limited research exists on sexual harassment in PSFs. Professional service firms such as the “Big 4” accounting firms and “Magic Circle” law firms are among “the most commonly recognized professional businesses” (9, p. 32) and play a vital role in the global economy. Furthermore, despite the influx of large numbers of women into the accounting and legal professions over the past couple of decades, these organizational sites have remained largely resistant to pressures of social inclusion, diversity, and equality. A large body of research shows how these PSFs have masculinized working practices and management cultures, remain horizontally and vertically segregated on gender lines, and reinforce existing gender inequalities (9–13). In contrast, few studies to date have examined sexual harassment in PSFs. A few older studies suggest that 30%−55% of female and about 10% of male lawyers experience workplace sexual harassment (14). Some more recent studies suggest that lawyers are more inclined to perceive the same behaviors as sexual harassment when the victim is a woman (15, 16).

Based on a random sample of 483 female members of the Association of International Certified Professional Accountants (AICPA), Stanko and Schneider (17) documented that 55% of women indicated that they knew women CPAs who had experienced sexual harassment while employed in public accounting. Thirty-seven percent of their respondents also reported having experienced sexual harassment in the accounting workplace. Recent anecdotal evidence also suggests that sexual harassment remains prevalent in accounting and law firms (18, 19). At the same time, many of these firms have actively promoted diversity initiatives over the last decade (20). One would hope that such initiatives have helped to reduce sexism and sexual harassment within these firms.

For the current study, survey data were gathered from 321 Belgian employees (155 women and 166 men) of global accounting and law firms. The prevalence of sexual harassment in PSFs based on traditional sexual harassment items and not-man-enough harassment items was reported (21). Correlations of respondents' experiences with workplace sexual harassment with perceived acceptance of sexual harassment by one's peers (social norms), personality traits, and demographic and job-related factors (age, sex, parental status, job level, and PSF type) were examined. Specifically, the current study addresses the following questions: How prevalent is sexual harassment in accounting and law firms, and what factors increase employees' risk of becoming victims of sexual harassment in such firms?

## 2. Materials and methods

### 2.1. Participants

Participants were 166 men and 155 women, aged 22 to 67 (*M* = 32.0, *SD* = 9.7), recruited through LinkedIn in April–May 2018. Participants had to reside in Belgium in order to be able to participate. Participants were not compensated for their participation. No sample size calculations were performed before the start of the study. The study was reviewed and approved by the Ethics Committee for Social and Humanities research of the University of Antwerp.

The majority (72.3%) of respondents reported being in a relationship. Most respondents (96.5%) described their sexual orientation as heterosexual. Respondents were at different levels in their organizational hierarchy, with 52 (16.2%) being at the executive level, 97 (30.2%) at the management level, 93 (28.9%) at the non-supervisory employee level, and 79 (24.6%) at entry level. The sample has 139 employees from accounting firms (66 men and 73 women) and 182 employees from law firms (100 men and 82 women).

### 2.2. Procedure and measures

All data were collected using an online survey. Participants completed a series of measures capturing workplace sexual harassment, Big 5 personality traits, and perceived norms about sexual harassment. All measures used in the present study were administered in the original English version. At the end of the survey, participants were asked to provide demographic information. In order to minimize social desirability bias, participants were asked about their experiences and opinions about (sexual) behaviors at work, purposely avoiding the term sexual harassment throughout the entire survey.

#### 2.2.1. Sexual harassment

To measure whether people had experienced sexual harassment at work, the Workplace Sexual Harassment scale (21) was used. This measure gauges two components of workplace sexual harassment, namely traditional sexual harassment, which encompasses sexist and sexual comments, unwanted sexual attention, and sexual coercion (e.g., “Forced themselves on you sexually”), and not-man-enough harassment, which encompasses behaviors that challenge a target's courage, strength, or toughness (e.g., “Said you were too sensitive”). Participants rated how often they had experienced certain situations at work during the past 24 months. The scale consists of 14 traditional sexual harassment and 5 not-man-enough harassment items, all rated on fully-labeled scales ranging from 0 (never) to 4 (most of the time). The internal consistency for this instrument was α = 0.84 for the present study. Cronbach's alpha are 0.82 and 0.70 for the traditional sexual harassment and the not-man-enough harassment subscales, respectively.

#### 2.2.2. Social norms

Perceived acceptance of sexual harassment by one's peers (social norms) was measured with six items (22, 23), asking participants to rate the extent to which other people would judge behavior to be acceptable on fully-labeled scales ranging from 1 (strongly disagree) to 7 (strongly agree). The items refer to behaviors such as “making sexual comments, jokes, gestures,” “showing, giving, sending, or leaving sexual pictures or text messages,” and “touching, grabbing, or pinching someone in a sexual way.” This measure captures the perceived rules or expectations about what behaviors are acceptable or common within a respondent's peer group. Cronbach's alpha for this scale is 0.79 for the present study.

#### 2.2.3. Big Five personality traits

To measure the Big Five personality traits (i.e., Extraversion, Agreeableness, Conscientiousness, Neuroticism, and Openness), the Ten Item Personality Inventory (TIPI) was used (22). The TIPI consists of 10 items (e.g., “Reserved, quiet”). For each item on the TIPI, participants rated the extent to which a set of personality traits apply to them using fully-labeled scales ranging from 1 (strongly disagree) to 7 (strongly agree). This short form of the Big Five strongly correlates with longer measures of Big Five personality (24). The TIPI has only two items for each domain, so within-scale inter-item correlations are appropriate for evaluating the internal consistency of each scale, rather than Cronbach's α coefficients. In the current study, the within-scale correlation coefficients between items in each pair range from *r* = 0.68 (Extraversion) to *r* = 0.21 (Agreeableness), resembling results from previous studies (24, 25).

#### 2.2.4. Sexist discrimination

Sexist discrimination was measured with four items (26), asking participants to rate the extent to which they had experienced certain events during the past year [e.g., “being treated unfairly by your employer, boss or supervisors because you are a (man/woman)”] on fully-labeled scales ranging from 1 (never) to 6 (almost all of the time). Cronbach's alpha for this scale was 0.88 for the present study.

## 3. Results

The overall prevalence of sexual harassment within PSFs in Belgium was first examined. The mean value of overall workplace sexual harassment is 1.33 (*SD* = 0.33) and is similar for women (*M* = 1.35, *SD* = 0.34) and for men (*M* = 1.32 , *SD* = 0.32), *t*_(320)_ = 0.804, *p* = 0.422, *d* = 0.10, 95% CI (0.14, 0.34). Mean values for traditional sexual harassment and not-man-enough harassment are 1.30 (*SD* = 0.34) and 1.42 (*SD* = 0.49), respectively. Traditional sexual harassment is similar for women (*M* = 1.29 , *SD* = 0.36) and for men (*M* = 1.31 , *SD* = 0.32), *t*_(320)_ = 0.490, *p* = 0.312, *d* = 0.06, 95% CI (−0.18, 0.30). Interestingly, however, women (*M* = 1.51 , *SD* = 0.49) in PSFs experience more not-man-enough harassment than men (*M* = 1.33, *SD* = 0.48), *t*_(320)_ = 2.930, *p* = 0.002, *d* = 0.36, 95% CI (0.12, 0.61). Further analyses showed that 76.6% of respondents (74.8% of women and 78.8% of men) said they experienced at least once or twice some form of traditional sexual harassment at work during the past 24 months, and 68.7% of respondents (80.2% of women and 57.6% of men) experienced some form of not-man enough harassment. Overall, 85.9% of respondents (88.5% of women and 83.3% of men) indicated to have experienced some form of sexual harassment at work during the past 24 months.

Moreover, half of the respondents (both women and men) had been in at least four different sexual harassment situations at least once or twice. Table 1 shows that the most common situations of sexual harassment were situations where someone “Made sexist comments or jokes?” (experienced by 72.7% of respondents, 59.5 and 65.9% of women and men, respectively), someone “Told sexual stories or jokes?” (experienced by 65.4% of respondents, 61.8 and 68.9% of women and men, respectively), and someone “Made you feel like you were not tough enough (for example, assertive, strong, or ambitious enough) for the job?” (experienced by 51.3% of respondents, 60.3 and 42.4% of women and men, respectively). Acts of severe physical sexual harassment (e.g., having been in a situation where someone “Forced themselves on you sexually?”) were relatively uncommon. Nevertheless, 10.3% of respondents (11.5% of women and 9.1% of men) had been in a situation in the past 24 months where someone at work “Attempted to establish a romantic or sexual relationship despite your efforts to discourage it?”

**Table 1:** Prevalence of sexual harassment in professional service firms (*N* = 321).

**Sexual harassment**	**Item**	**Total**	**Men**	**Women**
Traditional	Tried to draw you into a discussion of sexual matters?	27.9%	26.5%	29.0%
Traditional	Told sexual stories or jokes?	65.4%	68.9%	61.8%
Traditional	Displayed, used, or distributed sexual materials (pictures, stories, or pornography)?	16.7%	21.2%	12.2%
Traditional	Made sexist comments or jokes?	72.7%	65.9%	59.5%
Traditional	Gave you sexual attention?	26.2%	24.2%	28.2%
Traditional	Attempted to establish a romantic or sexual relationship despite your efforts to discourage it?	10.3%	9.1%	11.5%
Traditional	Pressured you to “play along” with sexual jokes and behavior?	13.4%	15.2%	12.2%
Traditional	Made you feel you needed to flirt with them to be treated well?	6.5%	4.5%	8.4%
Traditional	Touched your face, butt, thigh, or another “private” part of your body?	11.1%	6.8%	15.3%
Traditional	Exposed a private part of their body to you?	3.4%	6.1%	1.5%
Traditional	Forced themselves on you sexually?	1.5%	2.3%	0.8%
Traditional	Indicated there might be some reward or special treatment if you agreed to engage in sexual behavior?	0.4%	0%	0.8%
Traditional	Made you afraid that you would be penalized if you did not agree to engage in sexual behavior?	0.4%	0%	0.8%
Traditional	Treated you badly for refusing to have sexual relations with them?	1.2%	0.8%	1.5%
Not-man enough	Made you feel like you were not tough enough (for example, assertive, strong, or ambitious enough) for the job?	51.3%	42.4%	60.3%
Not-man enough	Called you a wimp, sissy, chicken, or some other name implying you are not courageous enough?	16.0%	15.9%	16.0%
Not-man enough	Implied they would admire you more if you were stronger or more athletic?	9.2%	12.1%	6.1%
Not-man enough	Teased you for being gullible or easily fooled?	32.4%	17.3%	37.4%
Not-man enough	Said you were too sensitive?	35.1%	20.5%	49.6%

The largest differences in situations experienced by women and men were “Made you feel like you were not tough enough (for example, assertive, strong, or ambitious enough) for the job?” (60.3% of women and 42.4% of men), “Someone teased you for being naive or easily fooled” (37.4% of women and 17.3% of men), and “Someone said you were too sensitive” (49.6% of women and 20.5% of men).

Next, correlates of sexual harassment were examined. To this end, linear regressions were ran with workplace sexual harassment, traditional sexual harassment, and not-man-enough harassment as the dependent variables (Table 2). As independent variables, personality traits, perceived social norms, and demographic and job-related factors (age, sex, parental status, job level, and PSF type) were included. These analyses provide some evidence for social norms' role in explaining the prevalence of workplace sexual harassment, especially traditional sexual harassment. Further, in line with the univariate results described earlier, these analyses show that women report experiencing more not-man-enough harassment than men. Finally, there is a negative association between job level and workplace sexual harassment, especially traditional sexual harassment (i.e., people higher up the organizational hierarchy experienced less harassment). The data do not provide evidence for associations between workplace sexual harassment and other factors included in the models.

**Table 2:** Predictors of self-reported experienced workplace sexual harassment (*N* = 321).

**Variable**	**Sexual harassment**		**Traditional SH**		**Not-man-enough**	
	***B*** **(SE)**	**95% CI**	***B*** **(SE)**	**95% CI**	***B*** **(SE)**	**95% CI**
Constant	1.41^**^ (0.25)	(0.92, 1.90)	1.40^**^ (0.26)	(0.89, 1.91)	1.43^**^ (0.36)	(0.73, 2.14)
Social norms	0.01^*^ (0.00)	(0.00, 0.02)	0.01^**^ (0.01)	(0.00, 0.02)	0.01 (0.01)	(−0.00, 0.02)
Extraversion	0.01 (0.01)	(−0.01, 0.02)	0.00 (0.01)	(−0.01, 0.02)	0.01 (0.01)	(−0.01, 0.03)
Agreeableness	−0.01 (0.01)	(−0.03, 0.02)	−0.02 (0.01)	(−0.04, 0.01)	0.02 (0.02)	(−0.02, 0.05)
Conscientiousness	0.00 (0.01)	(−0.02, 0.02)	0.00 (0.01)	(−0.02, 0.02)	−0.00 (0.02)	(−0.03, 0.03)
Neuroticism	−0.01 (0.01)	(−0.03, 0.01)	−0.00 (0.01)	(−0.02, 0.02)	−0.03 (0.02)	(−0.06, 0.00)
Openness	0.00 (0.01)	(−0.01, 0.03)	0.02 (0.01)	(−0.01, 0.04)	0.00 (0.02)	(−0.03, 0.03)
Sex	0.07 (0.05)	(−0.03, 0.2)	−0.02 (0.05)	(−0.08, 0.13)	0.19^**^ (0.07)	(0.05, 0.34)
Age	−0.01 (0.00)	(−0.02, 0.00)	−0.01 (0.00)	(−0.02, 0.00)	−0.01 (0.01)	(−0.02, 0.01)
Parental status	−0.07 (0.06)	(−0.19, 0.06)	−0.06 (0.07)	(−0.19, 0.08)	−0.10 (0.09)	(−0.28, 0.09)
Job level	−0.14^*^ (0.06)	(−0.26, −0.03)	−0.13^*^ (0.06)	(−0.2,. −0.12)	−0.16 (0.09)	(−0.33, 0.00)
PSF type	−0.06 (0.05)	(−0.16, 0.04)	−0.05 (0.05)	(−0.16. 0.05)	−0.09 (0.07)	(−0.23, 0.05)
*R* ^2^	0.31		0.27		0.24	

N = 321.

CI, confidence interval.

* p < 0.05.

** p < 0.01.

Further, logistic regressions (untabulated) were performed to ascertain the effects of personality traits, perceived social norms, and demographic and job-related factors on the likelihood that respondents experienced workplace sexual harassment (traditional sexual harassment, not-man-enough harassment, and overall sexual harassment). The logistic regression models were all statistically significant, *X*^2^ (11, *N* = 321) = 20.88, *p* = 0.04 (traditional sexual harassment), *X*^2^ (11, *N* = 321) = 28.11, *p* < 0.01 (not-man-enough harassment), *X*^2^ (11, *N* = 321) = 23.64, *p* < 0.01 (overall sexual harassment). The models explained, respectively, 11, 19, and 19% (Nagelkerke *R*^2^) of the variance in sexual harassment and correctly classified 80.1, 72.4, and 87.8% of the cases. Women were four times as likely to experience not-man-enough harassment [OR = 4.34, 95% CI (1.95, 9.62), *p* < 0.01] and three times as likely to experience overall sexual harassment [OR = 3.33, 95%CI (1.11, 9.95), *p* = 0.03]. The acceptance of sexual harassment by one's peers (social norms) was associated with an increase in the likelihood of traditional sexual harassment [OR = 1.10, 95% CI (1.01, 1.12), *p* = 0.03]. Overall sexual harassment was negatively associated with age [OR = 0.92, 95% CI (0.86, 0.99), *p* = 0.03] and was lower in accounting firms than in law firms [OR = 0.24, 95% CI (0.08, 0.72), *p* = 0.01].

Finally, whether sexual harassment was associated with sexist discrimination in the workplace was examined. Sexual harassment and sexist discrimination were found to be moderately positively correlated, *r*_(321)_ = 0.46, *p* < 0.01.

## 4. Discussion

Experiences of workplace sexual harassment were widespread in the current sample, with 88.5% of women and 83.3% of men reporting to have experienced at least once or twice some form of sexual harassment at work during the past 24 months. While these numbers are higher than those reported in most studies, they are comparable to those reported by Lonsway et al. (27) reporting on sexual harassment in law enforcement. A considerable body of research also shows that sexual harassment is more common in typically male-dominated professions (28, 29). Accounting and law have traditionally been male-dominated, and both professions are still characterized by skewed sex distributions at the top (i.e., those in power, the firms' partners, are men). The culture in these firms is often described as masculine (9) and discrimination and sexism are still rampant (11, 30). Hence, the current study's results align with research that links workplace sexual harassment with workplace culture (31).

The most frequent types of sexual harassment reported by respondents of the current study are examples of (verbal) forms of gender harassment. Gender harassment refers to behaviors that are not aimed at sexual cooperation but convey insulting, hostile, and degrading attitudes about women (traditional harassment) (33, p. 430) or about men (not-man-enough harassment) (22, p. 429). Instances of traditional harassment were experienced equally often by men and women in the current study. In contrast, not-man-enough harassment was much more frequently experienced by the women in the sample than by men. These results suggest that both men and women in PSFs experience enforcement of gender roles (albeit through somewhat different channels). It is also clear that such harassment creates a double bind for women (32). They are harassed and reminded that they are outsiders. However, they cannot resolve this by being more masculine, as they may experience even more negative repercussions if they do so (33). While most of the large accounting and law firms are now openly committed to diversity, equity, and inclusion, the results of the current study thus also highlight an important gap between formal policies and actual practices. The current study suggests that people experience more sexual harassment in environments with greater peer acceptance of such behavior. Other research also suggests that people are more likely to perpetuate such behaviors if they perceive acceptance by their peers (23). Therefore, it seems quintessential for firms to create working environments and cultures in which sexual harassment is clearly and unambiguously condemned and sanctioned. Recent studies have considered various potential interventions that could be applied as methods to reduce sexist and sexually aggressive attitudes among employees, including organizational policies and procedures, education, and training programs (34–36).

Respondents in my sample less frequently experienced severe physical sexual harassment. However, less severe but more frequent forms of gender harassment and more intense but less frequent forms of sexual harassment (i.e., unwanted sexual harassment or sexual coercion) have similar negative effects (e.g., on wellbeing) (5). All forms of sexual harassment are thus important and require attention from employers. Furthermore, while acts of sexual coercion were reported with relatively low frequency, no <10.3% of respondents (9.1% of men and 11.5% of women) nevertheless reported that they experienced (at least once during the past 24 months) an attempt from someone at work to establish a romantic or sexual relationship despite their efforts to discourage it, and 13.4% of respondents (15.2% of men and 12.2% of women) had been pressured to “play along” with sexual jokes or behavior.

There are a number of limitations to the current study. First, while the current study reports high numbers of sexual harassment in PSFs, the current data may still be an underestimation. If victims of sexual harassment are more likely to leave their firms or their professions altogether, this will lead to an underestimation of the actual levels of sexual harassment in PSFs. Conversely, however, the data may also overestimate the true levels of sexual harassment in PSFs because some of the behaviors on which respondents reported (e.g., having been drawn into a discussion of sexual matters) do not necessarily need to be experienced as negative by those involved. It would be interesting for future research to try to capture more accurately if people experienced all such occurrences as negative. At the same time, there is no room for discussion about the extent to which some of these behaviors are unwanted and unavoidably stressful or otherwise negative for their victims (e.g., attempts from someone to establish a romantic or sexual relationship despite efforts to discourage it, being pressured to “play along” with sexual jokes or behavior, sexual coercion). Second, the data for the current study came from a single country (Belgium) and were limited to employees of accounting and law firms. While accounting and law firms are the largest and most prominent PSFs, there are considerably few comparative studies providing insights into the similarities and differences of different types of PSFs, so the extent to which the results from the current study generalize to other PSFs (e.g., engineering consulting, architecture) is unknown. Third, the sample for the current study was too small to specifically investigate the harassment of LGBTQ+ people. Research has only started recently to explore the experiences of LGBTQ+ individuals in PSFs (37, 38). Future research in this area is encouraged to also specifically consider sexual harassment of LGBTQ+ individuals.

## 5. Conclusion

In this study, sexual harassment in accounting and law firms was examined. This study found that experiences of workplace sexual harassment are widespread in these firms. The most frequent types of sexual harassment reported were examples of (verbal) forms of gender harassment. These results align with research that links workplace sexual harassment with workplace culture. Results of the current study suggest that both men and women in PSFs experience enforcement of gender roles (albeit through somewhat different channels). Therefore, it seems quintessential for firms to create working environments and cultures in which sexual harassment is clearly and unambiguously condemned and sanctioned.

## Data availability statement

The raw data supporting the conclusions of this article will be made available by the authors, without undue reservation.

## Ethics statement

The studies involving human participants were reviewed and approved by Ethics Committee for Social and Humanities research (EASHW). The patients/participants provided their written informed consent to participate in this study.

## Author contributions

Conceptualization, study design, data collection, data analysis, and writing: KH.
